# Palatal vault morphometric analysis of the effects of two early orthodontic treatments in anterior open bite growing subjects: a controlled clinical study

**DOI:** 10.1186/s12903-021-01886-5

**Published:** 2021-10-11

**Authors:** Valeria Paoloni, Dimitri Fusaroli, Ludovica Marino, Manuela Mucedero, Paola Cozza

**Affiliations:** 1 Department of Systems Medicine, University of Rome “Tor Vergata”, Viale Oxford, 81, 00133 Rome, Italy; 2 Department of Dentistry UNSBC, Tirana, Albania; 3 UniCamillus-Saint Camillus International University of Health Sciences, Rome, Italy

**Keywords:** Anterior open bite, Early treatment, RME, Bite-Block, Quad Helix with crib, Palatal vault, GMM

## Abstract

**Background:**

The purpose of this study was to evaluate the palatal morphological changes in Anterior Open Bite (AOB) pre-pubertal subjects treated with Rapid Maxillary Expansion and Bite-Block (RME/BB) or Quad Helix with crib (QH/C) when compared with a Control Group (CG) by using Geometric Morphometric Analysis (GMM).

**Methods:**

AOB group (AOBG) included 30 subjects (20 females, 10 males, mean age 8.1 ± 0.8ys) with dentoskeletal AOB. AOBG was divided in two subgroups according to the treatment strategy: RME/BB group (RME/BBg) included 15 subjects (10 females, 5 males, QH/C group (QH/Cg) comprised 15 subjects (10 females, 5 males). The two subgroups were compared with a CG of 15 subjects (10 females, 5 males) matched for sex, age, vertical pattern, and observation period. Digital upper dental casts were collected before treatment (T1) and at the end of the active treatment (T2). Landmarks and semilandmarks were digitized on dental casts and GMM was applied. Procrustes analysis and principal component analysis (PCA) were performed.

**Results:**

At T2, RME/BBg when compared with QH/Cg evidenced no statistically significant differences. Instead, RME/BBg showed an increased maxillary transverse dimension and a decreased palatal depth when compared with CG. The comparison QH/Cg vs. CG demonstrated a slight transversal maxillary expansion.

**Conclusions:**

RME/BBg showed significant changes in the transversal and vertical dimensions with a clear maxillary expansion and a decrease of the palatal depth when compared with QH/Cg and CG. QH/Cg showed a significant slight maxillary expansion and no variation in vertical and sagittal planes when compared with CG.

## Background

The anterior open bite (AOB) is defined as an alteration in the vertical relationship between the maxillary and mandibular dental arches, characterized by a negative overbite that is a lack of contact between the upper and lower incisal edges in occlusion [1–3].

Epidemiological data report that 1 out of 20 subject presents open bite in mixed dentition [4, 5].

This malocclusion occurs because of cooperation of many etiological factors, both hereditary and environmental [6]. Increased vertical growth pattern and/or skeletal transverse discrepancy are correlated with genetic factor [7]; while environmental factors include extrinsic factors, such as sucking habits, which alter the vertical position of the incisors while skeletal relationships are normal [8]. However, in most cases, the distinction is not clear since this malocclusion presents both dental and skeletal components.

A broad diversity in terms of therapeutic approaches has been proposed in the early management of skeletal AOB.

Many Authors have emphasized that a skeletal open bite should be managed early in growing subjects by applying Rapid Maxillary Expander (RME) in association with a posterior Bite-Block (BB) [9–13].

Instead, Quad-Helix with crib (QH/C) is used in patients with dentoalveolar open bite, often related to sucking habits [9].

Patients treated before the pubertal peak exhibit significant and more effective long-term changes at the skeletal level in both maxillary and circummaxillary structures. When treatment is performed after the pubertal growth spurt, maxillary adaptations to expansion therapy shift from the skeletal level to the dentoalveolar level [14].

In literature, different studies examined the craniofacial effects of these two early treatment protocols on lateral cephalometric radiographs using bidimensional conventional analysis [9, 13, 15].

Only one study [8] evaluated the mandibular response and the mandibular morphometric changes to treatments using a different method of shape variations visualization represented by the Geometric Morphometric Method (GMM) [16–18] in OB subjects treated by RME/BB or by QH/C compared with a control group (CG).

As regards the palatal vault, GMM was used to analyse the morphological pre-treatment differences of this region in pre-pubertal subjects with open bite in comparison with a CG. The Authors concluded that subjects with open bite exhibited a significant constriction of the maxillary arch when compared with a CG without malocclusion, and that the morphological palatal shape variations in open bite patients were not influenced by the presence or absence of non-nutritive sucking habits [19].

To our knowledge, this is the first attempt to investigate the therapeutic effects on the palatal vault of two different early orthodontic treatments in growing subjects with AOB. This study wants to prove the importance of maxillary constriction in AOB subjects as an etiological factor.

Therefore, the aim of this study was to evaluate the palatal morphological changes in AOB pre-pubertal subjects after RME/BB and QH/C compared with an untreated AOB CG by using the GMM.

## Methods

A sample of 30 subjects with AOB (AOBG, 20 females, 10 males, mean age 8.1 ± 0.8 ys) was retrospectively collected from the archives of the Department of Orthodontics of the University of Rome “Tor Vergata”.

The project was approved by the ethical committee at the University of Rome “Tor Vergata” (protocol number 248/20) and all subjects’ parents signed the informed consent.

Each patient presented the following inclusion criteria: European ancestry (white), negative overbite, increased vertical dimension assessed on lateral cephalograms (SN^GoGN > 37°) [20], posterior transverse interarch discrepancy ≥ 3 mm [21], mixed dentition stage with fully erupted first permanent upper molars, prepubertal skeletal maturation (CS1–CS2) [22], good quality of pre-treatment (T1) and post-treatment (T2) records.

Exclusion criteria were: previous orthodontic treatment, multiple and/or advanced caries, appliance breakage, supernumerary teeth, tooth agenesis, cleft lip and/or palate, and other genetic diseases.

The initial AOBG was divided in two subgroups according to the treatment strategy: skeletal open bite subjects were treated by the RME in association with a posterior BB (RME/BBg), while dentoalveolar open bite subjects were treated using the QH/C (QH/Cg).

RME/BBg was composed of 15 subjects (10 F, 5 M; mean age 8.1 ± 0.9 ys); gQH/C enrolled 15 subjects (10 F, 5 M; mean age 8.1 ± 0.7 ys).

The T2-T1 time interval was in mean 1.5 ± 0.6 years for gRME/BB and 1.7 ± 0.6 years for gQH/C (Table 1).

**Table 1:** Demographics and statistical comparison of starting forms between Rapid Maxillary Expansion and Bite Block (RME/BB) group, Quad-Helix with Crib (QH/C) group and control group (CG) by means of ANOVA with Tukey post hoc tests (*p* < 0.05)

	RME/BB (n = 15, 10 f 5 m)		QH/C (n = 15, 10 f 5 m)		CG (n = 15, 10 f 5 m)		ANOVA	TUKEY post hoc tests (*p* value)		
Measurements	Mean	SD	Mean	SD	Mean	SD	*P* value	QH/C vs.RME *p* value	QH/C vs.CG *p* value	RME vs.CG *p* value
Age at T1 (years)	8.1	0.9	8.1	0.7	8.4	1.6	NS	NS	NS	NS
Age at T2 (years)	9.7	0.8	9.7	0.11	10.2	1.7	NS	NS	NS	NS
T1-T2 interval (years)	1.5	0.6	1.7	0.6	1.6	0.8	NS	NS	NS	NS

*SD* Standard Deviations, *NS* Not Significant

The AOBG was compared with a CG of 15 untreated AOB subjects (10 F, 5 M; mean age 8.4 ± 1.6 ys). The CG subjects were untreated AOB patients who refused treatment and they underwent a follow up until they decided to start the therapy. The CG matched with AOBG for chronologic age, presence of negative overbite, skeletal vertical dysplasia (increased vertical dimension as assessed on lateral cephalograms with SN^GoGN > 37°) [20], skeletal maturation at T1 and observation period. The T2-T1 time interval for CG was 1.2 ± 0.4 years.

### Treatment protocols

Each RME/BBg patient underwent the same treatment protocol with a “Butterfly” Rapid Maxillary Expander soldered to bands placed on the second deciduous molars or on the first permanent molars [23].

The expansion screw was activated once a day until the palatal cusps of the upper posterior teeth approached the buccal cusps of the lower posterior teeth, in overcorrection.

At the end of the active phase, the RME was left in place for 8 months to make stable the expansion achieved. After RME removal, no removable upper retainer was applied.

**Fig. 1:**
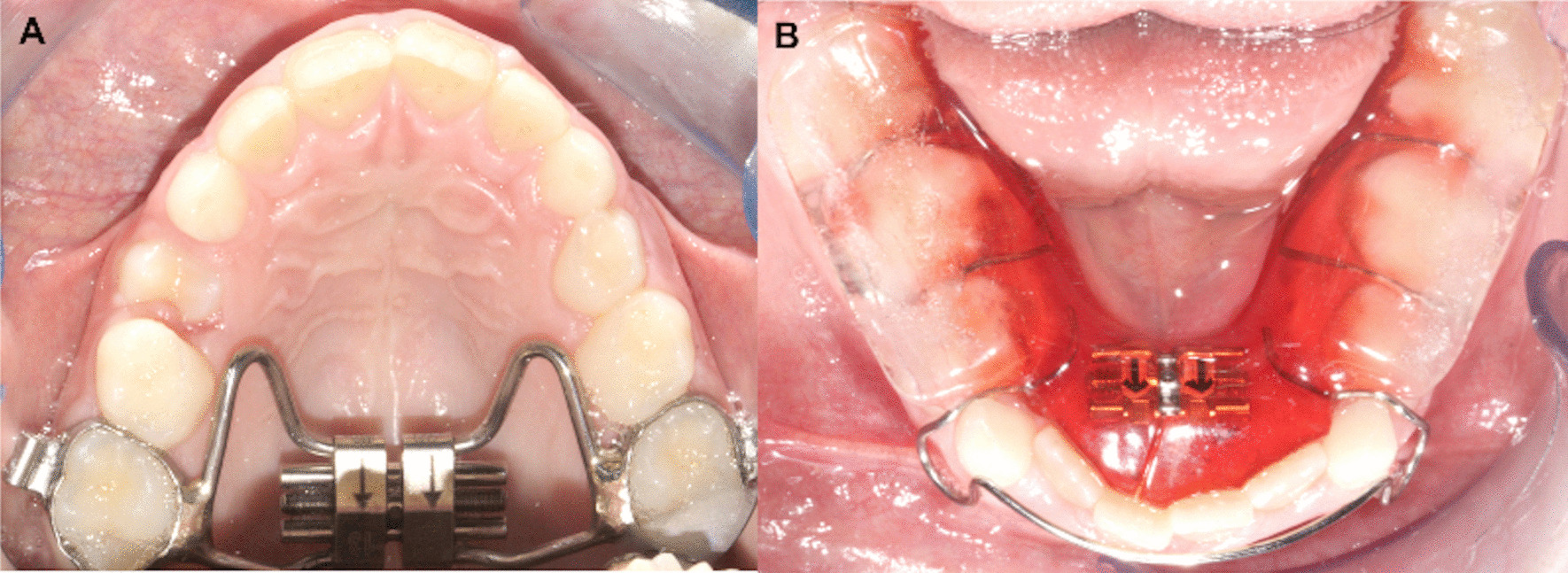
**a** Rapid maxillary expander. **b** Posterior bite block appliance

The BB is designed as a Schwarz device for the mandibular arch, with resin splints of 5 mm thickness in the posterior occlusal region (Fig. 1). The BB has been prescribed for 12 months to control the vertical dimension. Patients were instructed to wear the BB full time, 24 h a day, except for meals and for toothbrushing [24].

As any study involving a removable device, compliance varies among patients. Therefore, a single investigator conducted an interview with each patient to assess his/her collaboration. Compliance was assessed with a 3-point Likert-type scale (poor, moderate, good) [25]: poor compliance was reported when the patient wore BB at night only, moderate compliance occurred when the patient wore BB at night and during the day at home, and good compliance was assessed when the patient wore BB full-time, as suggested by the clinician [13].

QH/Cg used a QH/C made of 0.036-inch stainless steel wire, soldered to bands on the first permanent molars or on the second deciduous molars [26].

**Fig. 2:**
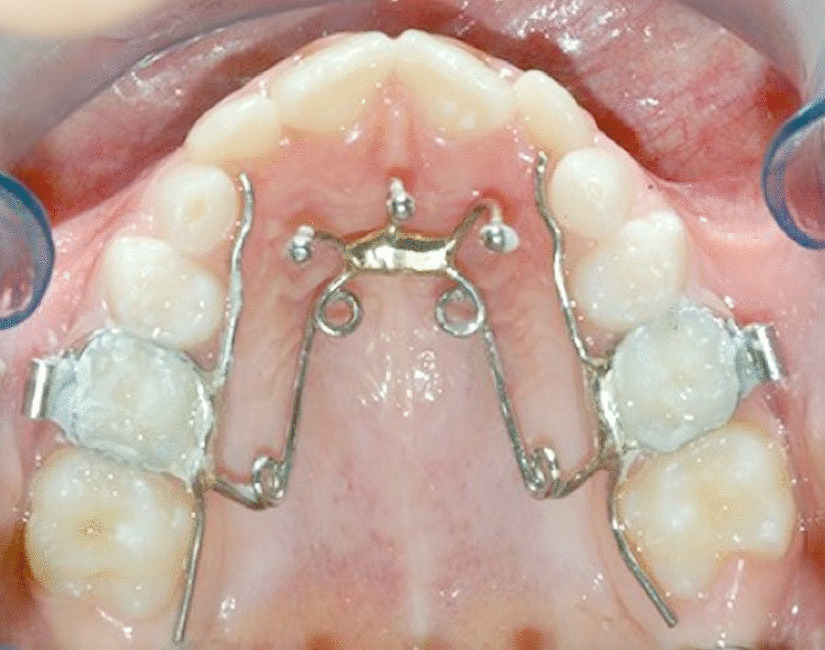
Quad Helix with crib

The crib was made of three spurs of 0.036-inch stainless steel wire positioned on the anterior bridge of the QH/C to avoid thumb sucking. The three segments are inclined lingually to prevent impingement on the sublingual mucosa (Fig. 2) [9, 27].

The therapeutic protocol required the activation of the QH/C of the transverse width of a molar. The device was reactivated once or twice during treatment to achieve overcorrection of the transverse relationships.

Two clinicians with similar experience for both the appliances (12–15 years) treated all the patients.

### Measurement protocol

In order to analyse the palatal shape, dental casts of the maxillary arches of all subjects were collected at the end of the active treatment (T2) and were scanned using an extraoral scanner with a reported accuracy of 20 μm (OrthoX scan, Dentaurum, Ispringen, Deutschland) and all models were exported in Standard Tesselation Language format (digital file .stl) [19].

3D GMM was chosen to fully study the palatal shape [17, 18, 28].

Viewbox 4 software (dHAL software, Kifissia, Greece) was used to digitize the post-treatment (T2) digital casts.

**Fig. 3:**
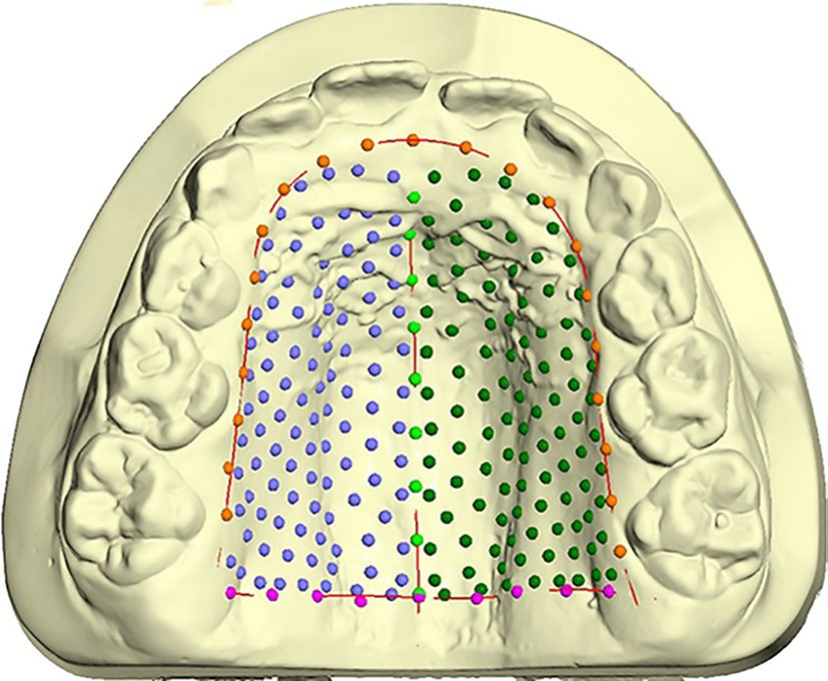
The three curves drawn on the digital dental casts. Green points: midsagittal suture; orange: perimeter of the dental arch on margin; pink: posterior border tangent to the distal surface of permanent first molars; dark green and blue: semilandmarks on the palatal surface

On each digital cast, landmarks were digitized to draw three curves and a total of 239 semilandmarks [29] were automatically obtained (Fig. 3).

The curves defined the palatal boundaries as: midsagittal suture (9 points); perimeter curve of the dental arch passing apical to the gingival sulci of each tooth (21 points); posterior curve passing from distal of the first permanent molars (9 points) [19].

The remaining points (semilandmarks) were placed uniformly on the palatal surface within the boundaries delimited by the three curves [30].

The averages of all the datasets of the palatal morphologies were calculated and these were used as a fixed reference (Procrustes’ average) to allow all the semilandmarks to slide and become more homologous among the different subjects, in order to minimize the Thin-Plate Spline (TPS) bending energy [18, 29, 31]. This procedure has been repeated twice.

## Statistical analysis

20 study casts were randomly selected and redigitized by the same trained operator (XX) two weeks later to determine the reliability of the method.

Random error was expressed as the distance between repeated digitisations in shape space compared with the total sample variance [16].

Procrustes superimposition was used to extract Procrustes’ coordinates for the shape description and principal component analysis (PCA) was performed to reveal the main patterns of palatal shape variation.

Procrustes distance among the groups means was used to evaluate the statistical differences among the groups at T2: RME/BBg versus QH/C; RME/BBg versus CG; QH/Cg versus CG. More than 10 000 permutations have been reported [18].

In the presence of normally distributed data, statistical inter-group comparisons for the T2 demographics data were performed using ANOVA with Tukey *post hoc* tests (*P* < 0.05).

## Results

At T1 RME/BBg patients presented a posterior transverse interarch discrepancy of 5.4 ± 0.5 mm, QH/Cg subjects showed a constricted maxillary arch discrepancy of 4.1 ± 0.4 mm while the CG of 4.9 ± 0.6 mm.

The analysis of compliance of the RME/BBg subjects for the use of BB, evidenced that no one had poor collaboration, 2 had moderate cooperation, and the remaining 13 patients had good compliance. As a result, cooperation was good in 86.7% of the patients.

At T2 in the QH/Cg and RME/BBg the overbite was greater than 0 mm in all the patients.

No statistically significant differences were found between the three groups in the analysis of the demographics data.

The mean random error of the 20 repeated digitisations for the geometric morphometric analysis, expressed as a percentage of total shape variance, was 2.9%.

**Fig. 4:**
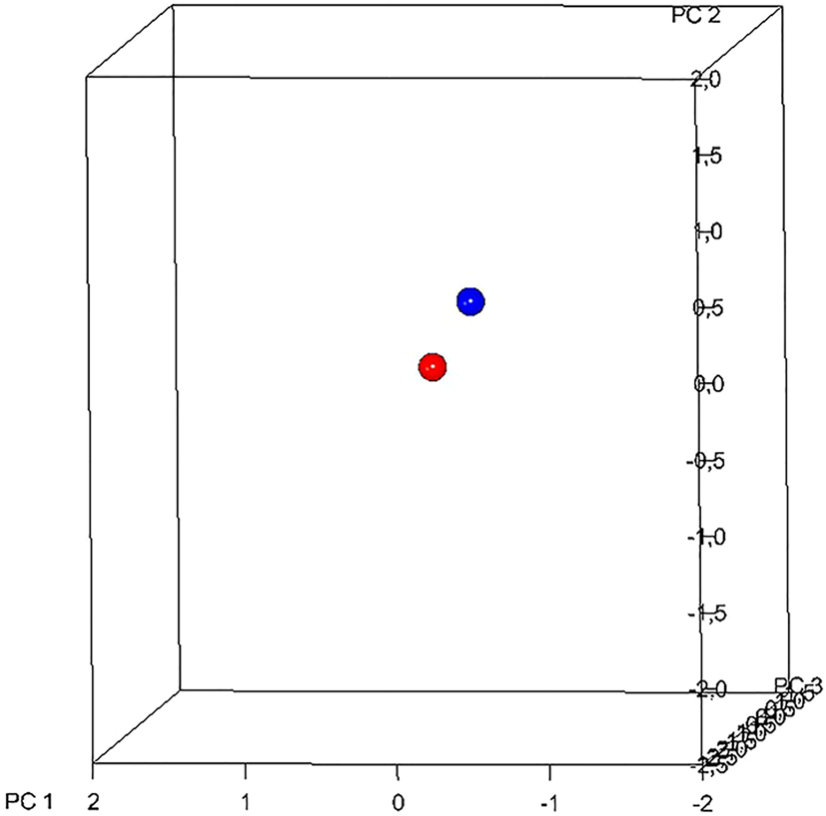
Plot of the distribution of shape average of RME/BBg (blue sphere) vs. QH/Cg (red sphere) in the craniofacial shape space with standard deviation at the end of the active treatment (T2)

**Fig. 5:**
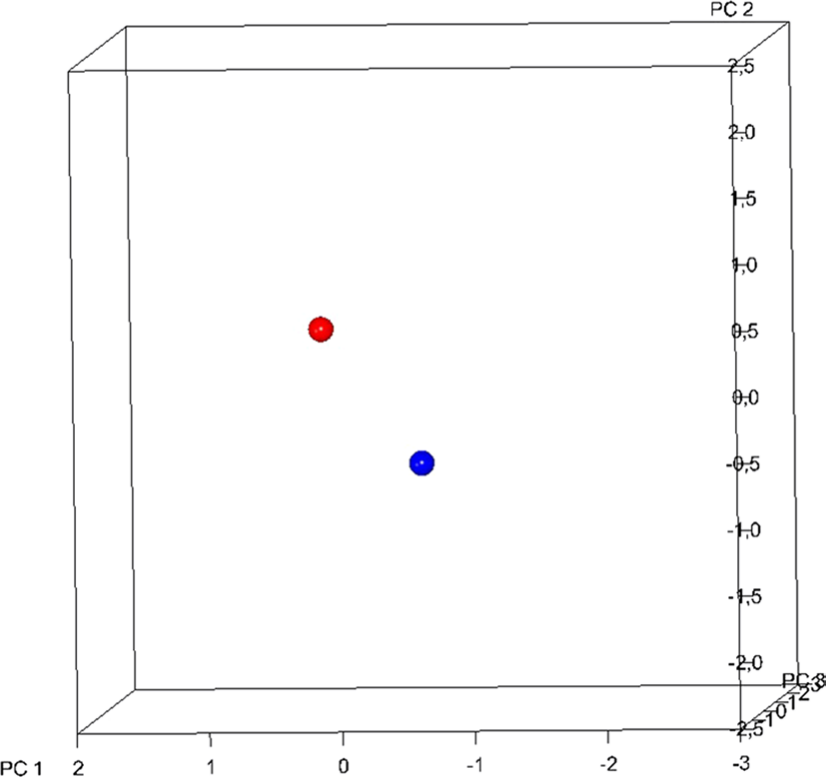
Plot of the distribution of shape average of RME/BBg (blue sphere) vs. CG (red sphere) in the craniofacial shape space with standard deviation at the end of the active treatment (T2)

**Fig. 6:**
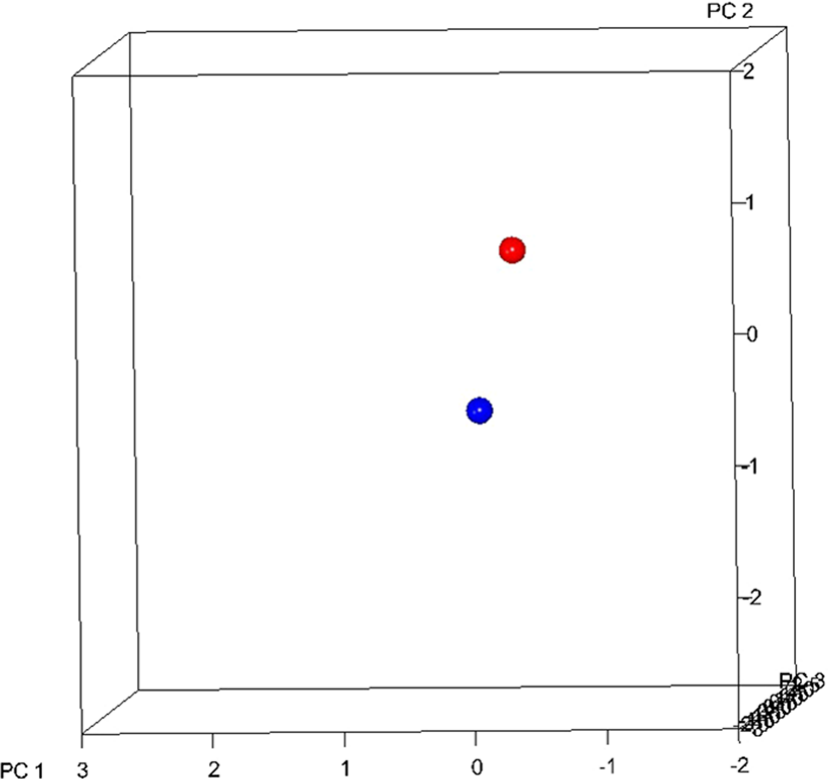
Plot of the distribution of shape average of QH/Cg (blue sphere) vs. CG (red sphere) in the craniofacial shape space with standard deviation at the end of the active treatment (T2)

For the variations in the palatal vault morphology, the comparison RME/BBg vs. QH/Cg showed no statistically significant changes at T2, while RME/BBg vs. CG and QH/Cg vs. CG showed a statistically significant difference (10 000 permutations; *p* = 0.69; *p* = 0.0093; *p* = 0.0075) (Figs. 4, 5 and 6). The first principal component (PC1) described the most important variance and was morphologically considered to be the most relevant.

**Fig. 7:**
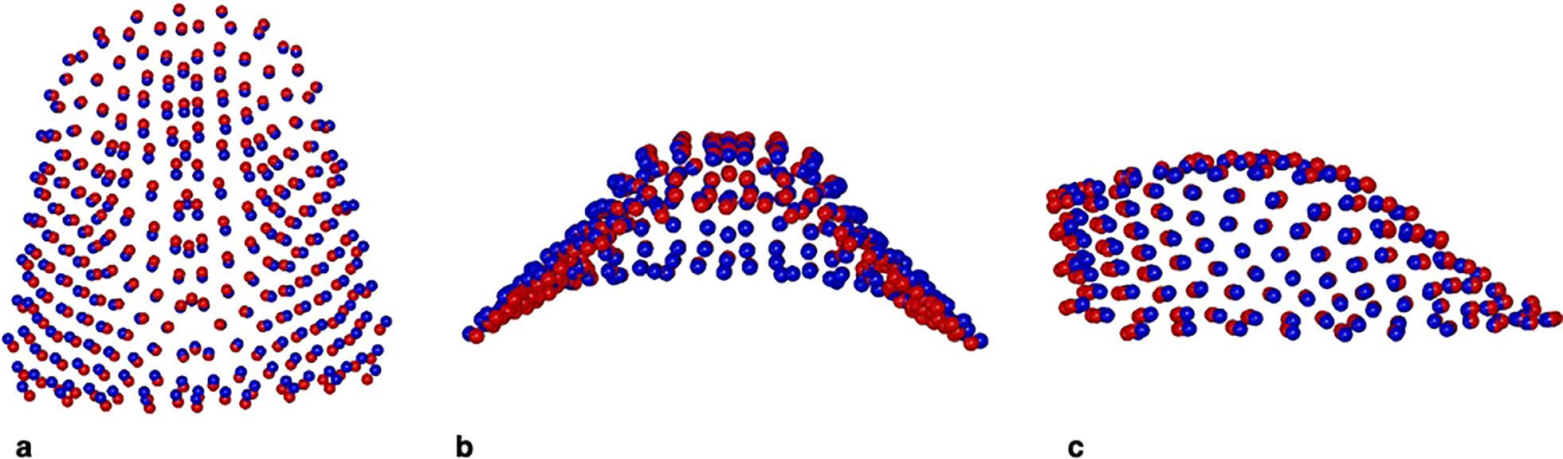
Morphological maxillary comparison between RME/BBg (blue) and QH/Cg (red) at the end of the active treatment (T2). **a** Global view from above. **b** Posterior view. **c** Sagittal view

PC1 RME/BBg vs. QH/Cg included the 43.4% of total shape variance (PC1: 43.4%; PC2: 18.5%; PC3: 9.0%; PC4: 6.7%). Figure 7 showed minor morphological differences between the palatal vaults average at the end of the two different treatments with a slight greater transversal expansion in RME/BBg, though no statistically significant.

**Fig. 8:**
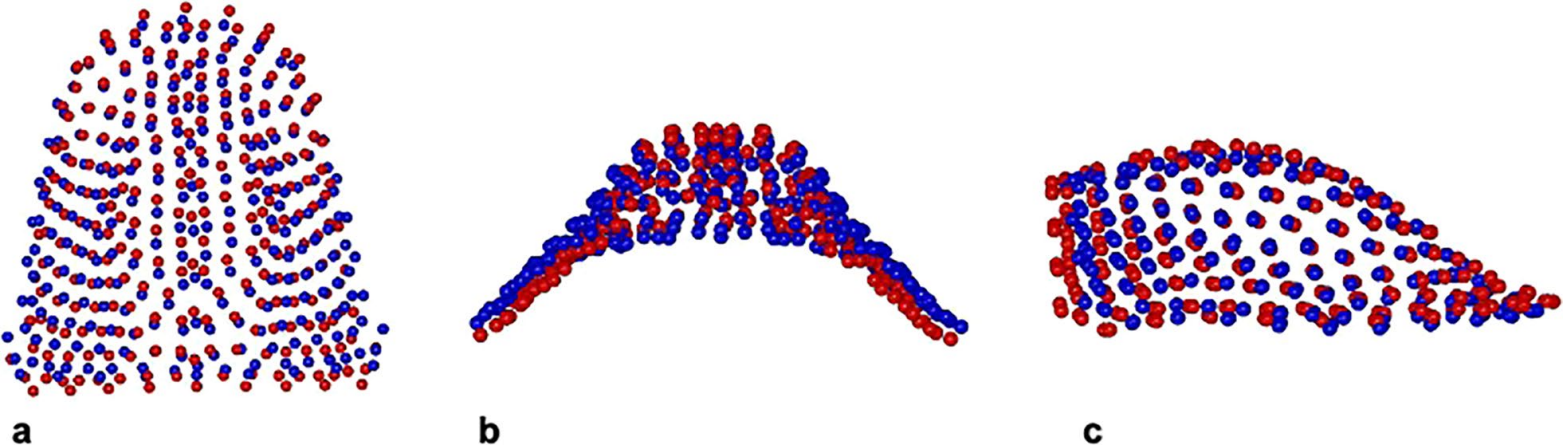
Morphological maxillary comparison between RME/BBg (blue) and CG (red) at the end of the active treatment (T2). **a** Global view from above. **b** Posterior view. **c** Sagittal view

By analysing RME/BBg and CG, PC1 variation defined the 30.8% of total shape variance (PC1: 30.8%; PC2: 19.2%; PC3: 19.2%; PC4: 10.8%; PC5: 6.4%). The palatal vault in RME/BBg was statistically significant more transversal expanded and less deep than in CG (Fig. 8).

**Fig. 9:**
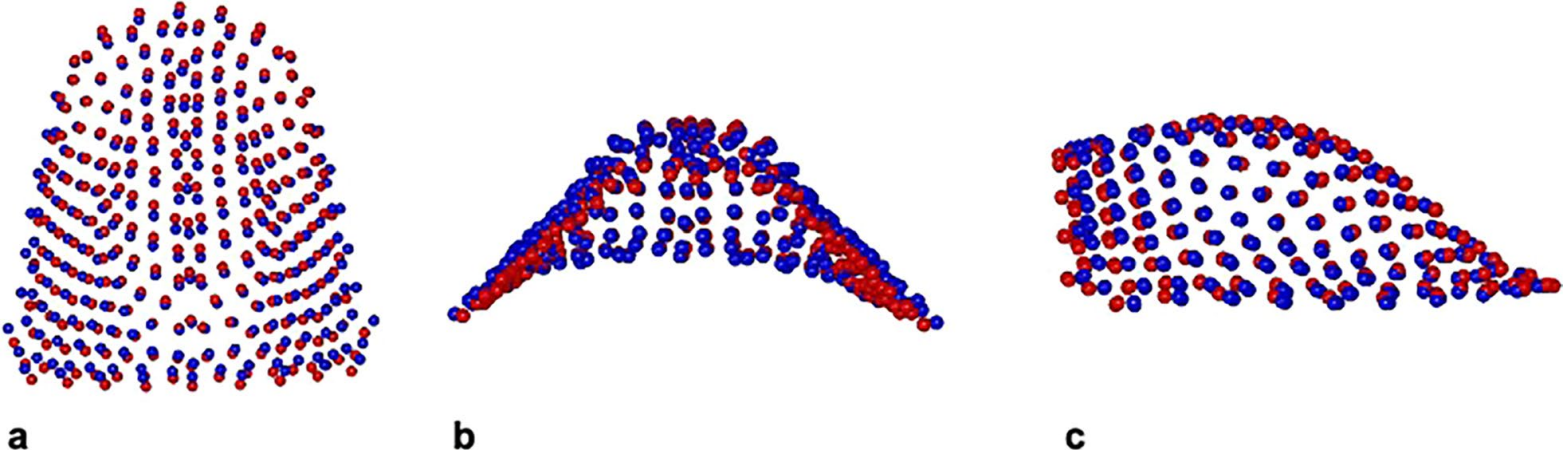
Morphological maxillary comparison between QH/Cg (blue) and CG (red) at the end of the active treatment (T2). **a** Global view from above. **b** Posterior view. **c** Sagittal view

By comparing QH/Cg and CG, PC1 variation characterized the 29.8% of total shape variance (PC1: 29.8%; PC2: 18.0%; PC3: 14.5%; PC4: 8.5%; PC5: 7.4%). The QH/Cg palatal vault was slightly statistically significant expanded in its transverse dimension than CG one, while there were no significant variations in maxillary depth (Fig. 9).

## Discussion

The purpose of the present study was to evaluate the morphological changes of the palatal vault in AOB growing subjects after two different early orthodontic treatments (RME/BB and QH/C) compared with an untreated AOB CG by using GMM.

In literature, the maxillary morphology of pre-treatment open bite subjects has been widely described. Several studies revealed the presence of a significantly narrower maxillary arch in these patients when compared with a control group [15, 32–34]. However, they used bidimensional analysis on dental casts as inter-canine and inter-molar widths, providing incomplete information about the tridimensional morphology of the palatal vault [35, 36].

Recently, GMM was proposed as a new method of comprehensive shape evaluation that can communicate even complex morphological changes much more effectively than coefficients that result from traditional morphometric analysis [16].

GMM shows shape changes not only in preselected areas (i.e., molars and canine transverse distance, palatal height, palatal depth), but virtually in any point of the surface where homologous landmarks and semilandmarks were positioned [37].

When using GMM, we renounce to have any information on size, as all the shapes are “averaged” and size information is left out of the Procrustes space. This can be seen as a disadvantage as only change in shape patterns can be outlined through GMM. Anyway, this limitation can turn into an advantage. In fact, there is no need to arbitrarily select a special part of the shape to be measured as all parts can be compared as far as a landmark fits the area. While looking at palates, we can get much more information through a GMM procedure rather than with standardized measures. Another important aspect is that in orthodontics we normally compare anatomical features between patients and controls, assuming that controls are more regular or “normal.” However, what can be considered normal or not normal, is controversial and of difficult interpretation. With GMM, variation of shapes just comes out from the population, considering all the aspects of the shape, without the need of pre-selecting some parts of the population. Variability analysis through PCA allows to determine shape patterns and can thereafter dictate which measures to take and not vice versa. When pre-selecting patients with different anatomical features (like in the example of palates collected from oral breathers and standard breathers), GMM has the role to underline the source of differences between the two samples. If the samples are really different as for their space entities, they should appear clusterized, as at least the group with pathologic problem (oral breathers) represents an extreme of the population [37].

Using the means of GMM, Krey KF. et al. [38] observed that patients with skeletal AOB present a short mandibular ramus due to growth deficit. In addition, significant differences were found in terms of maxillary vertical development in AOB subjects when compared with untreated AOB subjects. However, the authors focused on adult patients excluding growing ones, while our study included pre-pubertal subjects.

Freudenthaler J. et al. [39] used GMM to evaluate the role of craniofacial complex in different malocclusions in a sample of patients from 7 to 39 years, showing that AOB subjects have the maxilla tilted upwards while the mandible downwards.

In 2019, Laganà G. et al. [19] analysed the morphological palatal vault shapes’ changes in growing AOB subjects, with or without referred prolonged sucking habits, compared with a control group with good occlusion through the means of GMM. They found that AOB subjects showed a significant constriction of the maxillary arch when compared with the CG and that the morphological palatal shape variations in AOB subjects were not influenced by the presence or absence of non-nutritive sucking habits.

However, the mentioned studies described the palatal morphological characteristic of the AOB subjects and they did not observe the morphological changes occurring in these patients after orthodontic treatment.

Recently, one study [8] evaluated the morphometric changes in AOB growing subjects after two different orthodontic treatment (RME/BB or QH/C) compared with an untreated AOB control group, by using conventional cephalometry and GMM. The authors analysed only the effects of these treatments on the mandible. They found that that RME/BB subjects showed significant changes in the vertical orientation of the mandibular ramus with a tendency for the mandible to rotate counterclockwise when compared with QH/C subjects and CG, resulting in a divergence reduction of the mandibular and occlusal planes. In contrast, the QH/C protocol did not affect the mandibular morphology [8].

To our knowledge, this is the first attempt to study the morphometric changes of the palatal vault in AOB growing subjects after two different early therapeutic protocols (RME/BB and QH/C) compared with an untreated AOB CG by using GMM.

According to Laganà et al. [19], our study group was composed by AOB subjects without distinguishing the dentoalveolar or skeletal etiological nature of the malocclusion.

The initial AOBG was divided into two subgroups according to the treatment strategy adopted: subjects with skeletal OB were treated by RME/BB, subjects with dentoalveolar AOB were treated by QH/C. Then a CG, that matched the AOBG for chronologic age, malocclusion and skeletal maturation, was collected.

As suggested by Paoloni et al. [30], the palatal vault, analysed through the means of GMM, was assessed up to the gingival margin in order to eliminate the influence of dental inclination and position on the alveolar bone.

This investigation showed that RME/BBg when compared with QH/Cg at T2 had no statistically significant differences. This result may be explained because every treatment strategy, chosen for each patient, was the proper one to correct the AOB malocclusion. Therefore, a correct occlusion was obtained because the etiological factor was removed.

The results of this study showed that at the end of active therapy (T2) the RME/BBg presented a palatal vault more expanded and less deep than the CG.

These findings agree partially with the ones present in literature on patients with maxillary constriction [40–44] and confirm that RME significantly increases transversal dimensions of the palatal vault.

However, to our knowledge, no study associated the RME to a decrease in the palatal depth. On the contrary, Bruder C. et al. [44] demonstrated that maxillary constricted patients treated by RME have no vertical alteration of the palate. This result is in contrast with the one of our study. The difference is in the treatment protocol. Our AOB patients were treated with RME and BB that controlled the vertical dimension reducing the extrusion of maxillary and mandibular molars and applying an intrusive force on the teeth and consequently on the bones [13].

When comparing QH/Cg vs. CG, the GMM analysis showed significant differences in the morphometric shape of the palatal vaults. QH/Cg was slightly expanded than CG ones, while there were no variations in maxillary depth. The entity of the transverse expansion was inferior to the one obtained by RME/BB therapy. This result agrees with several studies [27, 45−47] that demonstrated the transversal variation obtained by the use of the quad-helix in growing patients.

Mucedero et al. [9] showed also that the QH/C protocol produced a clinically significant downward rotation of the palatal plane evaluated on the lateral cephalometric radiographs. Meanwhile, our study showed no 3D morphological variation in vertical and sagittal direction of the palatal vault in QH/Cg vs. CG because the QH/C induced a bodily downward rotation of the maxilla with no evidence in the GMM.

Our results demonstrated the correlation between palatal morphology and AOB malocclusion and highlighted the clinical need to manage AOB early in growing subjects by treating maxillary constriction in order to obtain an easier resolution of the malocclusion [9–13].

## Conclusions

- GMM is a helpful way to visually represent and depict palatal vault changes in growing patients with AOB malocclusion after early treatment.
- RME/BBg showed significant changes in the transversal and vertical dimensions with a clear maxillary expansion and a decrease of the palatal depth when compared with QH/Cg and CG.
- In RME/BBg, the decrease of the palatal depth contributed to AOB correction and was influenced by the use of BB.
- QH/Cg showed significant changes only in the transverse dimension with a slight maxillary expansion and no variation in vertical and sagittal planes when compared with CG.
- QH/C induced a bodily downward rotation of the maxilla which contributed to AOB correction.

## Data Availability

Data are available on justified request to the authors.

## Abbreviations

AOB: Anterior open bite
RME: Rapid maxillary expander
BB: Bite-Bock
RME/BB: Rapid maxillary expansion and bite block
QH/C: Quad Helix with crib
CG: Control group
GMM: Geometric morphometric analysis
AOBG: AOB group
RME/BBg: RME/BB group
QH/Cg: QH/C group
PCA: Principal component analysis

## References

[1] Subtelny JD, Sakuda M (1964). Open-bite: diagnosis and treatment. Am J Orthod Dentofac Orthop.

[2] Worms FW, Meskin LH, Isaacson RJ (1971). Open-bite. Am J Orthod.

[3] Ngan P, Fields HW (1997). Open bite: a review of etiology and management. Pediatr Dent.

[4] Kelly JE, Sanchez M, Van Kirk LE. An assessment of the occlusion of the teeth of children 6-11Years, United States. Vital Health Stat. 1973;11(130):1–60. 25209689

[5] Proffit WR, Fields HW, Moray LJ (1998). Prevalence of malocclusion and orthodontic treatment need in the United States: estimates from the NHANES III survey. Int J Adult Orthodon Orthognath Surg.

[6] Cozza P, Baccetti T, Franchi L, Mucedero M, Polimeni A (2005). Sucking habits and facial hyperdivergency as risk factors for anterior open bite in the mixed dentition. Am J Orthod Dentofacial Orthop.

[7] Grippaudo C, Oliva B, Greco AL, Sferra S, Deli R. Relationship between vertical facial patterns and dental arch form in class II malocclusion. Prog Orthod. 2013;14:43. 10.1186/2196-1042-14-43. 10.1186/2196-1042-14-43 PMC4384936 24326093

[8] Lione R, Fusaroli D, Mucedero M, Paoloni V, Pavoni C, Cozza P. Changes in mandibular shape after early treatment in subjects with open bite: a geometric morphometric analysis. Eur J Orthod. 2020;15:cjz104. 10.1093/ejo/cjz104. 10.1093/ejo/cjz104 31942983

[9] Mucedero M, Franchi L, Giuntini V, Vangelisti A, McNamara JA, Cozza P (2013). Stability of quad-helix/crib therapy in dentoskeletal open bite: a long-term controlled study. Am J Orthod Dentofacial Orthop.

[10] Rodrigues de Almeida R, Ursi WJ. Anterior open bite. Etiol Treat. Oral Health. 1990;80(1):27–31. 2388763

[11] English JD (2002). Early treatment of skeletal open bite malocclusions. Am J Orthod Dentofacial Orthop..

[12] Luzzi V, Guaragna M, Ierardo G, Saccucci M, Consoli G, Vestri AR, Polimeni A (2011). Malocclusions and non-nutritive sucking habits: a preliminary study. Prog Orthod.

[13] Mucedero M, Fusaroli D, Franchi L, Pavoni C, Cozza P, Lione R (2018). Long-term evaluation of rapid maxillary expansion and bite-block therapy in open bite growing subjects: a controlled clinical study. Angle Orthod..

[14] Baccetti T, Franchi L, Cameron CG, McNamara JA (2001). Treatment timing for rapid maxillary expansion. Angle Orthod.

[15] Cozza P, Mucedero M, Baccetti T, Franchi L (2005). Early orthodontic treatment of skeletal open-bite malocclusion: a systematic review. Angle Orthod..

[16] Papagiannis A, Halazonetis DJ (2016). Shape variation and covariation of upper and lower dental arches of an orthodontic population. Eur J Orthod..

[17] Mitteroecker P, Gunz P (2009). Advances in geometric morphometrics. J Evol Biol.

[18] Klingenberg CP (2013). Visualizations in geometric morphometrics: how to read and how to make graphs showing shape changes. Hystrix, the Italian J Mammal.

[19] Laganà G, Di Fazio V, Paoloni V, Franchi L, Cozza P, Lione R. Geometric morphometric analysis of the palatal morphology in growing subjects with skeletal open bite. Eur J Orthod. 2019;41(3):258–63. 10.1093/ejo/cjy055. 10.1093/ejo/cjy055 30102344

[20] Riolo ML, Moyers RE, McNamara JA, McNamaraHunter WS (1974). An Atlas of Craniofacial Growth: Cephalometric Standards from The University School Growth Study, The University of Michigan.

[21] Tollaro I, Baccetti T, Franchi L, Tanasescu CD (1996). Role of posterior transverse interarch discrepancy in Class II, Division 1 malocclusion during the mixed dentition phase. Am J Orthod Dentofacial Orthop.

[22] McNamara JA, Franchi L (2018). The cervical vertebral maturation method: a user’s guide. Angle Orthod.

[23] Cozza P, Giancotti A, Petrosino A (1999). Butterfly expander for use in the mixed dentition. J Clin Orthod..

[24] Lione R, Kiliaridis S, Noviello A, Franchi L, Antonarakis GS, Cozza P (2017). Evaluation of masseter muscles in relation to treatment with removable bite-blocks in dolichofacial growing subjects: a prospective controlled study. Am J Orthod Dentofacial Orthop.

[25] Slakter MJ, Albino JE, Fox RN, Lewis EA (1980). Reliability and stability of the orthodontic Patient Cooperation Scale. Am J Orthod.

[26] Marino A, Laganà G, Mucedero M, Polimeni A, Cozza P (2003). Terapia del morso aperto da parafunzione: Quad Helix con griglia. Mondo Ortodontico.

[27] Cozza P, Giancotti A, Rosignoli L (2000). Use of a modified Quad Helix in early interceptive treatment. J Clin Orthod..

[28] Polychronis G, Halazonetis DJ (2014). Shape covariation between the craniofacial complex and first molars in humans. J Anat.

[29] Parcha E, Bitsanis E, Halazonetis DJ (2017). Morphometric covariation between palatal shape and skeletal pattern in children and adolescents: a cross-sectional study. Eur J Orthod..

[30] Paoloni V, Lione R, Farisco F, Halazonetis DJ, Franchi L, Cozza P (2017). Morphometric covariation between palatal shape and skeletal pattern in Class II growing subjects. Eur J Orthod..

[31] Bookstein FL (1997). Landmark methods for forms without landmarks: morphometrics of group differences in outline shape. Med Image Anal..

[32] Warren JJ, Bishara SE (2002). Duration of nutritive and nonnutritive sucking behaviors and their effects on the dental arches in the primary dentition. Am J Orthod Dentofacial Orthop..

[33] Aznar T, Galán AF, Marín I, Domínguez A (2006). Dental arch diameters and relationships to oral habits. Angle Orthod..

[34] Katz CR, Rosenblatt A, Gondim PP (2004). Nonnutritive sucking habits in Brazilian children: effects on deciduous dentition and relationship with facial morphology. Am J Orthod Dentofacial Orthop.

[35] Gracco A, Malaguti A, Lombardo L, Mazzoli A, Raffaeli R (2010). Palatal volume following rapid maxillary expansion in mixed dentition. Angle Orthod..

[36] Primožic J, Baccetti T, Franchi L, Richmond S, Farčnik F, Ovsenik M (2013). Three-dimensional assessment of palatal change in a controlled study of unilateral posterior crossbite correction in the primary dentition. Eur J Orthod.

[37] Huanca Ghislanzoni L, Lione R, Cozza P, Franchi L (2017). Measuring 3D shape in orthodontics through geometric morphometrics. Prog Orthod..

[38] Krey KF, Dannhauer KH, Hierl T (2015). Morphology of open bite. J Orofac Orthop.

[39] Freudenthaler J, Čelar A, Ritt C, Mitteröcker P (2017). Geometric morphometrics of different malocclusions in lateral skull radiographs. J Orofac Orthop..

[40] Garrett BJ, Caruso JM, Rungcharassaeng K, Farrage JR, Kim JS, Taylor GD (2008). Skeletal effects to the maxilla after rapid maxillary expansion assessed with cone-beam computed tomography. Am J Orthod Dentofacial Orthop..

[41] Gohl E, Nguyen M, Enciso R (2010). Three-dimensional computed tomography comparison of the maxillary palatal vault between patients with rapid palatal expansion and orthodontically treated controls. Am J Orthod Dentofacial Orthop.

[42] Christie KF, Boucher N, Chung CH (2010). Effects of bonded rapid palatal expansion on the transverse dimensions of the maxilla: a cone-beam computed tomography study. Am J Orthod Dentofacial Orthop.

[43] Lione R, Franchi L, Fanucci E, Laganà G, Cozza P (2013). Three-dimensional densitometric analysis of maxillary sutural changes induced by rapid maxillary expansion. Dentomaxillofac Radiol.

[44] Bruder C, Ortolani CLF, Lima TA, Artese F, Faltin Junior K (2019). Evaluation of palate area before and after rapid maxillary expansion, using cone-beam computed tomography. Dental Press J Orthod..

[45] Boysen B, La Cour K, Athanasiou AE, Gjessing PE (1992). Three-dimensional evaluation of dentoskeletal changes after posterior cross-bite correction by quad-helix or removable appliances. Br J Orthod.

[46] Bench RW (1998). The quad helix appliance. Semin Orthod.

[47] Sollenius O, Golež A, Primožič J, Ovsenik M, Bondemark L, Petrén S (2020). Three-dimensional evaluation of forced unilateral posterior crossbite correction in the mixed dentition: a randomized controlled trial. Eur J Orthod..

